# Ultrasonic elastography and conventional ultrasound in the diagnosis of thyroid micro-nodules

**DOI:** 10.12669/pjms.35.6.777

**Published:** 2019

**Authors:** Jinming Wang, Wenbing Wei, Rui Guo

**Affiliations:** 1 Jinming Wang Department of Ultrasound, Binzhou People’s Hospital, Shandong, 256610, China; 2 Wenbing Wei Department of Ultrasound, Binzhou People’s Hospital, Shandong, 256610, China; 3 Rui Guo Department of Ultrasound, Binzhou People’s Hospital, Shandong, 256610, China

**Keywords:** Ultrasonic elastography, Conventional ultrasound, Thyroid micro-nodules

## Abstract

**Objective:**

To investigate the clinical value of conventional ultrasound, ultrasound elastography and conventional ultrasound combined with ultrasound elastography in differential diagnosis of benign and malignant thyroid micro-nodules.

**Methods:**

Eighty-six patients who were found with thyroid micro-nodules with the maximum diameter no more than 10 mm in the physical examination in our hospital from June 2015 to December 2017 were selected, and 102 nodules were included. All patients were confirmed with thyroid micro-nodules by puncture or surgical pathology and underwent conventional ultrasound and ultrasound elastography. Taking the surgical pathological results as a control, the value of conventional ultrasound, ultrasound elastography and conventional ultrasound combined with ultrasound elastography in differential diagnosis of benign and malignant thyroid micro-nodules were compared. A receiver operating characteristic (ROC) curve was drawn, and the area under the ROC curve was calculated.

**Results:**

One hundred and two thyroid nodules were detected by surgical pathology, including 75 benign nodules (73.53%) and 27 malignant nodules (26.47%). The sensitivity and diagnostic accordance rate of ultrasound elastography were significantly higher than those of conventional ultrasound in the diagnosis of thyroid microcarcinoma, and the missed diagnosis rate of ultrasound elastography was significantly lower than that of conventional ultrasound; the difference was statistically significant (P<0.05). However, the difference between the two methods was not statistically significant in terms of diagnostic specificity and misdiagnosis rate (P>0.05). The areas under the ROC curve in the diagnosis of benign and malignant thyroid nodules by conventional ultrasound and ultrasound elastography were 0.735 and 0.743 respectively.

**Conclusion:**

Conventional ultrasound can be used as a routine examination technique in differential diagnosis of benign and malignant thyroid nodules, while ultrasound elastography can improve the sensitivity and diagnostic rate in the diagnosis of thyroid micro carcinoma. It can help to reduce the clinical missed diagnosis, which also can be be used as an effective supplement for conventional ultrasound.

## INTRODUCTION

Thyroid nodules are clinically common endocrine system diseases, which mainly refer to abnormal proliferation and formation of masses with abnormal hardness and structure in the thyroid gland.^1^,^2^ A study pointed out that the incidence of thyroid nodules in adults could be as high as 40% and there were benign and malignant nodules.^3^ However, due to the small volume of thyroid micro-nodules, they are difficult to reach during physical examination. Besides, it lacks typical clinical symptoms and is often associated with Hashimoto’s disease and nodular goiter, which leads to high missed diagnosis and misdiagnosis rate; therefore, efficient and accurate judgment of benign and malignant thyroid nodules has drawn much clinical attention.^4^,^5^ At present, conventional ultrasound is still the main method for identifying benign and malignant thyroid nodules.^6^,^7^ However, the phenomenon that the same disease may have different images and the same image may indicate different diseases in ultrasonographic images of benign and malignant thyroid nodules increases the difficulty of accurate diagnosis.^8^ As a new ultrasound technique, ultrasonic elastography can reflect the hardness of tissues by evaluating deformation degree of normal or diseased tissues after external force, which makes up the weakness of traditional imaging methods in acquiring tissue hardness information, and it provides a new means for identification of benign and malignant nodules.^9^ However, ultrasonic elastography grading can be affected by many factors and has some limitations in clinical applications.^10^ This study aimed to evaluate the clinical value of conventional ultrasound and ultrasonic elastography in the differential diagnosis of benign and malignant thyroid nodules.

## METHODS

Eighty-six patients who were found having thyroid micro-nodules with the maximum diameter no more than 10 mm in the physical examination in our hospital between June 2015 and December 2017 were selected. The volume of samples was determined using the formula: n=2*[(α+β)σ/δ]^2. In the formula, δ stands for the required distinction degree, σ stands for the overall standard deviation, α and β stand for the u values of α and β, which can be checked out by the row of degree of freedom υ=∞- in the t critical value table.α can be unilateral or bilateral, while β only takes the unilateral value.

There were 48 females and 38 males and their ages ranged from 25 to 67 years (43.4±10.8 years). All patients underwent conventional ultrasound and ultrasonic elastography. All patients underwent surgical resection or biopsy and histopathological examination. This study was approved by the ethics committee of our hospital and all patients were informed and volunteered to participate in the study.

Diagnostic criteria referred to the relevant standards in the Chinese Expert Consensus for Diagnosis and Treatment of Thyroid Micropapillary Carcinoma (2016),^11^ i.e., having hard micro-nodules with good mobility and without pains under press, a fibrovascular axis in the center of tumor, obvious cell specificity, and ground-glass-like appearance in some nuclei.

First-onset patients who had solid nodules and two lobes of the thyroid gland with the maximum diameter less than 10 mm, were in accordance with thyroidectomy indications, had complete medical history and basic data, were conscious and had no language barrier were included. Pregnant or breast feeding women or patients with liver, kidney, heart and brain dysfunction, obvious acoustic shadowing after calcification around nodules or lesions unable to be zoomed more than 2 times during ultrasound elastography were excluded.

### Ultrasound examination

In the conventional ultrasound examination, patients were guided to lie in a supine position, and their heads were kept slightly reclined and necks were fully exposed. A Siemens Acuson S2000 color Doppler ultrasound apparatus which was equipped line array probe and had frequency between 5 and 10 MHz was used in continuous scanning of longitudinal, transverse and oblique sections to observe the location, size, morphology, boundary, internal echo and calcification of lesions and whether there were cervical lymph nodes nor not. Then the internal and peripheral blood flows of the lesion were observed through color Doppler flow imaging. In the case of specific examination, the blood flow velocity and the angle of the blood flow sampling line was reasonably controlled, and an angle of 60° was appropriate. The image was finally recorded and stored.

In the ultrasonic elastography examination, the body position and instrument used were the same as the conventional ultrasound examination. The ultrasonic elastography mode was adopted, the region of interest centered on the nodule was selected, and generally the region of interest was zoomed to two to three times that of the nodule. Then the probe was kept perpendicular to the skin, and the large blood vessels, trachea and coarse calcification of the neck were kept away from. Examination started to stable ultrasonic elastography images when the quality index on the display was more than 60 and the image quality was stable with good repeatability, and the images were saved.

### Ultrasound image analysis

The conventional ultrasound scoring criteria were based on the thyroid nodule scoring method recommended by Stacul et al.^12^ Nodules with regular morphology were given 0 point, those with irregular morphology were given two points, and those between them were given one point. Nodules with clear boundary were given 0 point, those with unclear boundary were given two points, and those between them were given one point. Nodules with complete acoustic halo were given 0 point, and those with no acoustic halo or incomplete acoustic halo was given one point. Nodules with cystic internal echo or mostly cystic internal echo was given 0 point, those with iso-echo, slightly high echo or mixed echo was given one point, and those with low echo and extremely low echo was given two points. Nodules with no calcification were given 0 point, those with coarse calcification were given one point, those with minor calcification were given two points, and those with two kinds of calcification were also given two points. Nodules with aspect ratio more than one were given 0 point, while those with aspect ratio no less than 1 was given one point. When the score of a thyroid nodule was four or more than four points, it was considered as malignant. When the score of a thyroid nodule was three or less than three points, it was considered as benign. Lesion hardness classification based on elastic image referred to the grading standard of thyroid nodules proposed by Zhou et al.^13^ the higher the hardness of nodule, the higher the grade and the larger the blue area in the nodule. Liquid nodular tissue whose elastic image showed typical red, green and blue was evaluated as grade 0. Green nodules and surrounding tissues was evaluated as grade I. Nodules which were dominated by green (50% < green < 90%) was evaluated as grade II. Nodules which showed messy blue-green mosaic or were dominated by blue (50% < blue < 90%) was evaluated as grade III. A nodule which was dominated by blue (blue > 90%) was evaluated as grade IV. As to the diagnosis standard, nodules with grade 0 ~ II were evaluated as benign, while those grade III ~ IV were evaluated as malignant.

### Statistical analysis

A database was built by Microsoft Excel 2007, and statistical analysis was performed by SPSS 20.0. The enumeration data was expressed by % and processed by chi-square test. The receiver operating characteristic (ROC) curve was used for evaluating the diagnostic value of the two methods. P<0.05 meant that difference was statistically significant.

## RESULTS

**Fig. 1:**
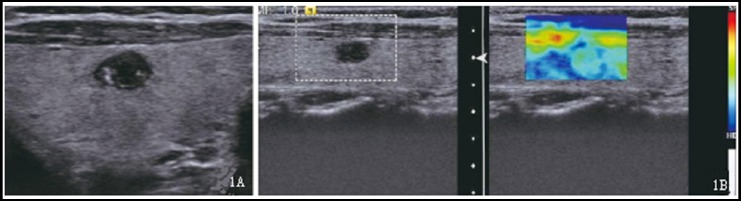
Conventional ultrasound and elastography images of nodular goiter. ***Note:*** Central nodules in the right lobe of the thyroid gland, 1A: conventional ultrasound examination found clear boundary (0 point), extremely low echo (2 points), and internally scattered, dotted strong echoes (2 points), and Stacul score: 4 points; 1B: grade II elastography imaging, pathological result: nodular goiter.

**Fig. 2:**
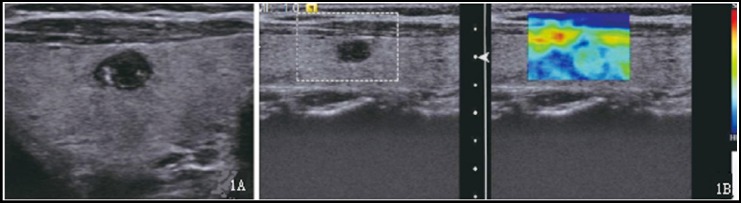
Conventional ultrasound and elastography images of papillary thyroid carcinoma. ***Note:*** Dorsal nodules in the middle of the thyroid gland, 2A: unclear boundary (1 point), internal iso-echo (1 point), wide or narrow incomplete acoustic halo (2 points), aspect ratio more than 1 (1 point), and internally scattered small strong echoes (2 points), Stacul score: 7 points; 2B: Grade W elastography imaging, pathological result: papillary thyroid carcinoma.

All patients were diagnosed by surgical pathology, and 102 thyroid nodules were detected. The pathological results were as follows. There were 75 benign nodules, 40 of which were nodular goiters, 28 were thyroid adenomas, and 7 were thyroid gland hyperplastic nodules. There were 27 malignant nodules, 19 of which were papillary adenomas, 7 were follicular adenomas, and 1 was medullary thyroid tumor. Fig. 1 and 2 show conventional ultrasound and elastography images of malignant and benign thyroid nodules.

Of the 27 malignant nodules confirmed by postoperative pathological diagnosis, 20 were diagnosed as malignant by conventional ultrasound, and the remaining 7 were diagnosed as benign. Of the 75 benign nodules confirmed by postoperative pathological diagnosis, 14 were diagnosed as malignant by conventional ultrasound, and the remaining 61 were diagnosed as benign.

Of the 27 malignant nodules confirmed by postoperative pathological diagnosis, 24 were diagnosed as malignant by ultrasound elastography, and the remaining three were diagnosed as benign. Of the 75 benign nodules confirmed by postoperative pathological diagnosis, eight were diagnosed as malignant by ultrasound elastography, and the remaining 67 were diagnosed as benign.

**Fig. 3:**
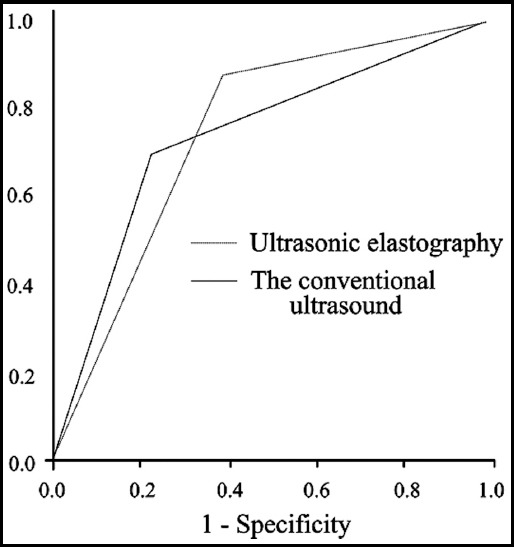
ROC curves of the conventional ultrasound and ultrasonic elastography in the diagnosis of benign and malignant thyroid micro-nodules.

According to the results of conventional ultrasound and ultrasonic elastography, the sensitivity, specificity, , missed diagnosis rates and diagnostic accordance rates of the two ultrasound diagnosis methods for thyroid micro carcinoma were calculated and compared taking the pathological diagnosis results as the gold standard. The results showed that the sensitivity and accurate diagnostic rate of ultrasound elastography in the diagnosis of thyroid microcarcinoma were significantly higher than those of the conventional ultrasound diagnosis, and the missed diagnosis rate of ultrasound elastography was significantly lower than that of the conventional ultrasound; differences were statistically significant (P<0.05). There was no significant difference between the two methods in terms of specificity and misdiagnosis rate (P>0.05, Table-I). The areas under the ROC curve of the conventional ultrasound and ultrasound elastography in the diagnosis of benign and malignant thyroid nodules were 0. 735 and 0.743 respectively (Fig. 3).

**Table-I:** Comparison of the efficacy of two ultrasound methods in the diagnosis of thyroid microcarcinoma (%).

Methods	Sensitivity	Specificity	Misdiagnosis Rate	Missed Diagnosis Rate	Diagnostic Accordance Rate
Conventional Ultrasound	74.1	81.3	18.7	25.9	79.4
Ultrasonic Elastography	88.9	89.3	10.7	11.1	89.2
X^2^	4.375	2.508	2.508	4.375	6.358
P	0.037	0.113	0.113	0.037	0.011

## DISCUSSION

In recent years, with the increasing awareness of thyroid disease and the widespread application of high-frequency ultrasound, the detection rate of thyroid nodules has increased from 20% to 76%, and most nodules had a diameter no more than one cm.^14^ A study has shown that some malignant thyroid micro-nodules could invade the capsule and surrounding tissues and cervical lymph node metastasis or even distant metastasis might occur.^15^ If patients with malignant nodules are not treated in time, endocrine and respiratory system functions will be affected, and in severe cases, it can cause cancer and even threaten patients’ life.

At present, conventional ultrasound is often used in the diagnosis of benign and malignant thyroid nodules. It has the advantage of simple, non-invasive, safe and repeatable operation. Moreover, ultrasonography can exclude malignant nodules at an early stage, showing a high diagnostic value. Conventional high-frequency ultrasound can clearly display nodules with a diameter less than 5 mm and accurately determine the position, number and size of nodules.^16^,^17^ However, conventional ultrasound still has limitations, that is, it cannot identify the hardness of nodules, which leads to a high misdiagnosis rate. In some cases, the pathological structure of nodules is more complicated, and conventional ultrasound images lack specificity under such a condition.^18^ Therefore there are many dissents on the diagnosis of thyroid micro-nodules with the conventional ultrasound, and how to improve the accuracy of diagnosis has been a key problem concerned in clinics.

Ultrasonic elastography is a new type of examination method developed based on conventional ultrasound. On the basis of conventional ultrasound diagnosis, different images can be formed under the influence of vibration and external force based on the difference in elastic index between tissues. The color code is used for hardness grading, and the pathological type of thyroid is judged according to the color coverage; in this way, the benign and malignant nodules can be effectively identified.^19^,^20^ The grade of hardness of benign nodules was lower, and the difference between benign nodules and conventional tissues in hardness was small. Most nodules in this study were at grade 1-2. Microcalcification is often seen in the malignant nodule tissues, and moreover significantly increased hardness, adhesion between the lesion and the surrounding tissues and decreased mobility can also be observed, which makes the hardness grade of malignant nodules higher than that of benign ones. In this study, the malignant nodules were mostly at grade 4-5. In the diagnosis of benign and malignant thyroid nodules, ultrasonic elastography images can be superimposed with conventional ultrasound images in the analysis of the hardness grading of thyroid nodules to reduce the misdiagnosis and missed diagnosis of conventional ultrasound and make the diagnosis more precise. It can provide precise guidance for formulation of clinical treatment schemes.^21^ Liu et al. pointed out that conventional ultrasound as a basic evaluation method and ultrasonic elastography as a new ultrasound technique both played a role in the diagnosis of benign and malignant thyroid nodules.^22^ Jin et al. found that ultrasonic elastography could improve the diagnostic sensitivity and accordance rate of thyroid microcarcinoma and reduce missed diagnosis and it could be used as a supplement to conventional ultrasound.^23^ The results of the present study showed that the sensitivity and diagnostic accordance rate of ultrasound elastography in the diagnosis of small cancers were significantly higher compared with those of conventional ultrasound gray-scale imaging and color Doppler ultrasound diagnosis, which could reduce clinical missed diagnosis, improve the specificity of diagnosis, and reduce misdiagnosis rate; therefore it could be used as an important supplement for conventional ultrasound diagnosis. Moreover it was found that ultrasonic elastography had relatively high missed diagnosis and misdiagnosis rates, which might be that the large size of nodules, internal hemorrhage of the nodules and calcification affected the elasticity score. Scacchi et al. pointed out that ultrasonic elastography combined with conventional high-frequency ultrasound had high sensitivity and accuracy in the diagnosis of benign and malignant thyroid nodules. Therefore, the examination method should be selected clinically according to the specific conditions, and combined diagnosis can also be adopted.^24^

## CONCLUSION

Conventional ultrasound can be used as a routine examination method in the diagnosis of thyroid micro-nodules. Ultrasound elastography can be used as a necessary supplement for conventional ultrasound, and the combination of them can improve diagnosis levels of thyroid micro-nodules.

### Authors’ Contribution:

**JMW:** Study design, data collection and analysis.

**WBW & RG:** Manuscript preparation, drafting and revising.

**JMW:** Review and final approval of manuscript.
